# Supplementary material for: Gut microbiota profile in CDKL5 deficiency disorder patients
Source: Sci Rep. 2024 Mar 28;14:7376. doi: 10.1038/s41598-024-56989-0 (PMC10978852; doi:10.1038/s41598-024-56989-0)
Supplement: Supplementary file 1 — Supplementary Information. [file 41598_2024_56989_MOESM1_ESM.pdf]

Table S1- Nutritional values of the enrolled subjects

	CDD (mean ± SD)		HD (mean ± SD)		*p*-Value (CDD *vs* HC)		Reference Values (LARN)	
**Variable**	**Children**	**Adults**	**Children**	**Adults**	**Children**	**Adults**	**Children**	**Adults**
Energy (kcal)	1065.4 ± 682.4	1280.7 ± 107.0	1504.6 ± 405.5	1630.9 ± 275.3	0.02	0.10	750-1860 kcal/day	1240-1910 kcal/day
Proteins (g)	41.9 ± 14.3	54.8 ± 4.8	51.8 ± 18.8	74.8 ± 16.2	0.35	0.10	9-50 (g/day)*	43-50 (g/day)*
Proteins (%E)	16.5 ± 3.9	17.6 ± 0.4	13.7 ± 1.4	19.0 ± 3.4	0.12	0.48	0.72-0.82 (g/kg/day)*	0.71 %*
Total Carbohydrates (g)	155.1 ± 1 39.1	186.3 ± 21.7	205.8 ± 35.2	177.0 ± 53.4	0.01	0.73		
Total Carbohydrates (%E)	49.8 ± 12.0	56.0 ± 5.2	53.54 ± 6.8	41.8 ± 9.6	0.35	0.10	45%-60% E**	45%-60% E**
Sugars (g)	40.0 ± 17.5	50.4 ± 7.9	76.0 ± 21.0	54.1 ± 33.1	<0.01	0.86	<15% E	
Starch (g)	51.2 ± 82.1	47.0 ± 25.8	48.5 ± 16.0	60.0 ± 42.0	0.07	0.73		
Fiber (g)	12.7 ± 13.7	17.6 ± 5.0	13.0 ± 6.6	15.8 ± 8.6	0.21	0.86	8.4 g/1000 Kcal	12.6-16.7 g/1000 Kcal
Soluble Fiber (g)	1.2 ± 0.8	1.9 ± 0.6	1.5 ± 1.0	1.2 ± 0.9	0.44	0.21		
Insoluble Fiber (g)	2.9 ± 2.1	5.2 ± 2.3	4.2 ± 3.7	4.8 ± 4.5	0.49	0.73		
Fats (g)	37.3 ± 12.1	37.6 ± 6.1	43.2 ± 5.7	58.9 ± 15.5	0.31	0.04	20%-40% E**	20%-40% E**
Saturated Fats (g)	12.8 ± 6.2	10.5 ± 3.6	17.7 ± 4.0	22.5 ± 3.6	0.20	<0.01	<10% E	<10% E

CDD, CDKL5-deficiency disorder; HC, heathy control; E, energy; *AR, Average Requirement; **RI, recommended intake.

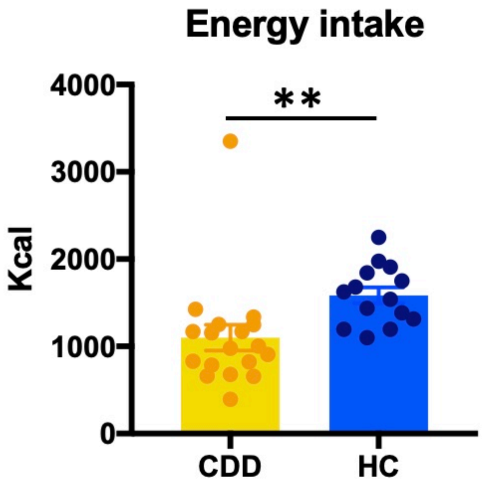

Suppl. Fig. 1. Food diary analysis.
The enrgy intake quantified as Kcal consumed/day was significantly decreased in CDD patients with respect to HC (Unpaired t-test, **p=0.002). The percentage of lipid, protein, carbohydrate and fiber consumed/day were not different. Circles represent single subject. Error bars represent SEM

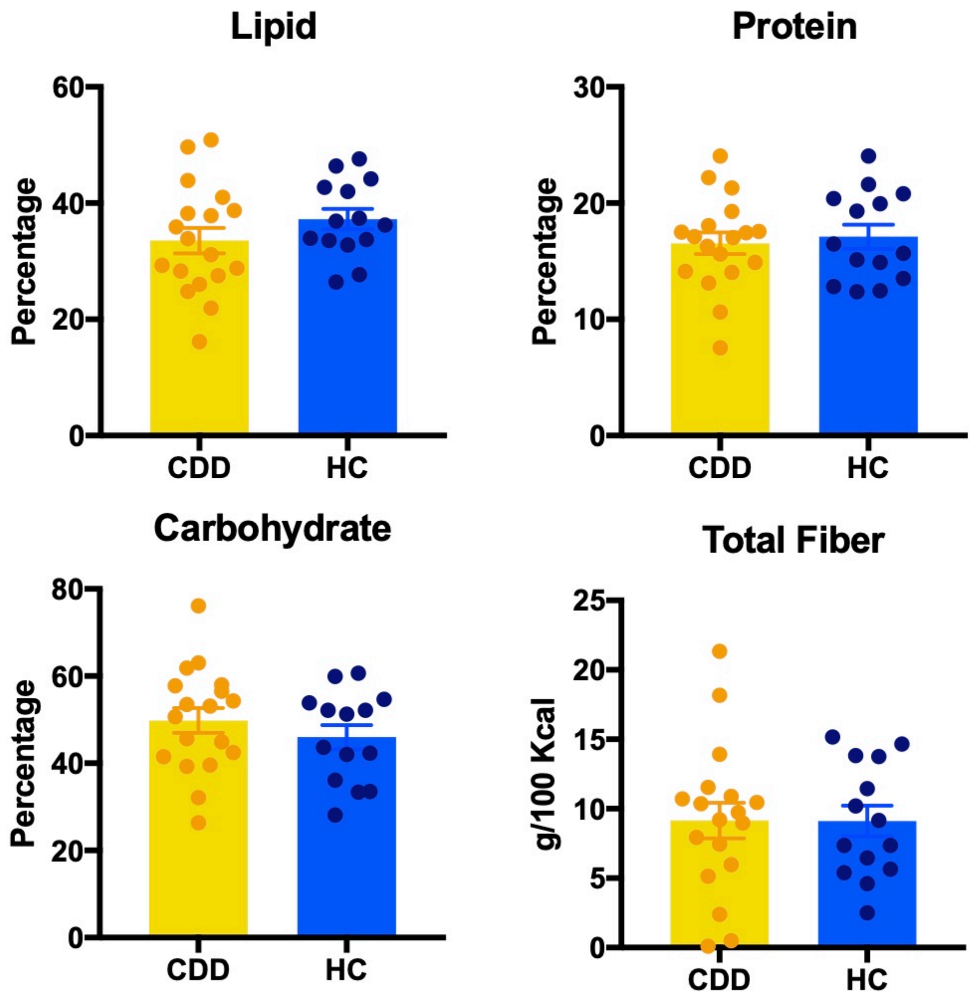

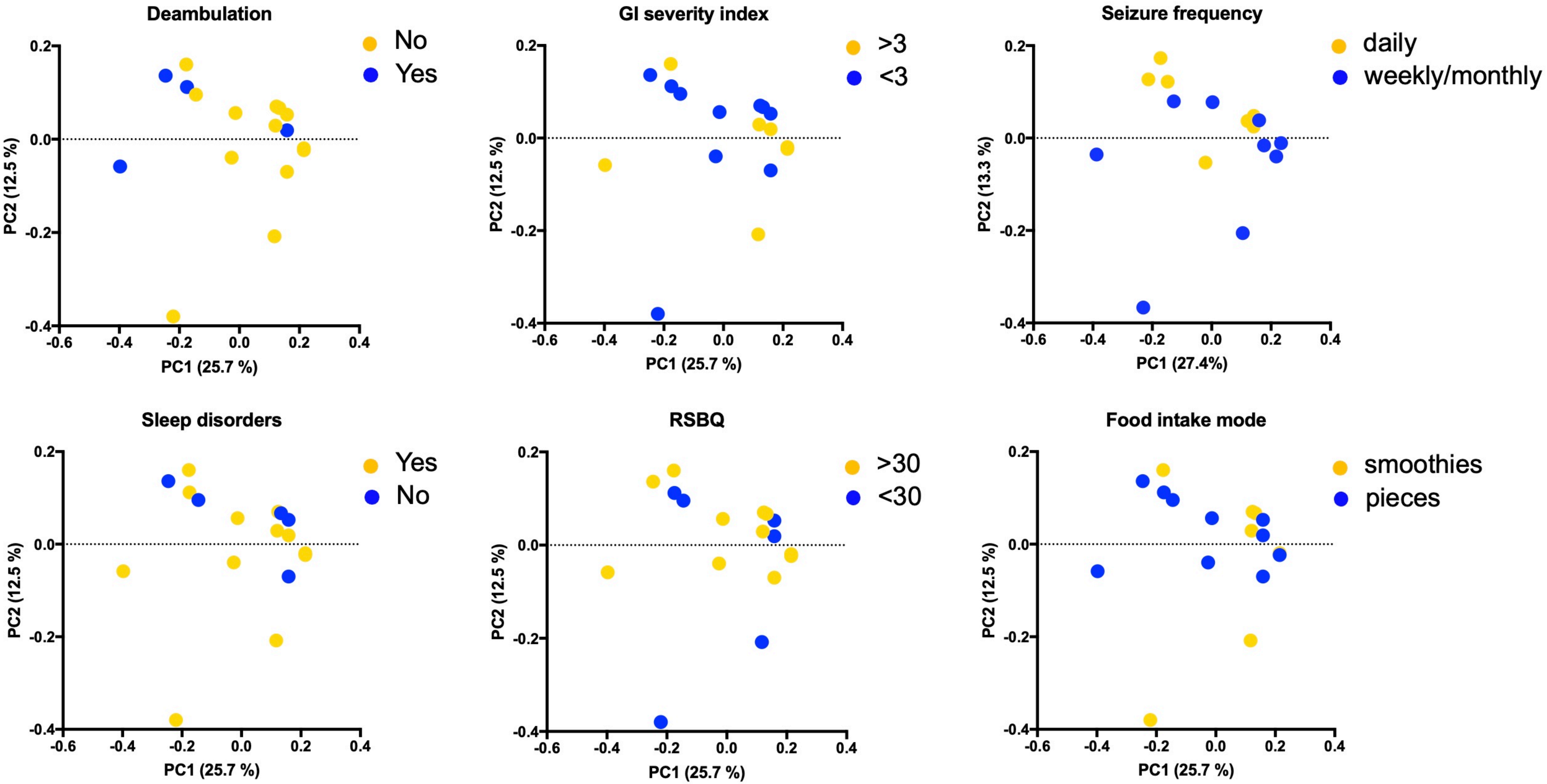

Suppl. Fig. 2. Beta-diversity in CDD patients. Patients were grouped based on deambulation capacity, GI severity index, the frequency of seizure episodes, the presence of sleep disorders, the score obtained in the RSBQ, and the mode of food intake. Principal coordinate analysis of Unweighted Unifrac Distances.
Circles represent single subjects.
